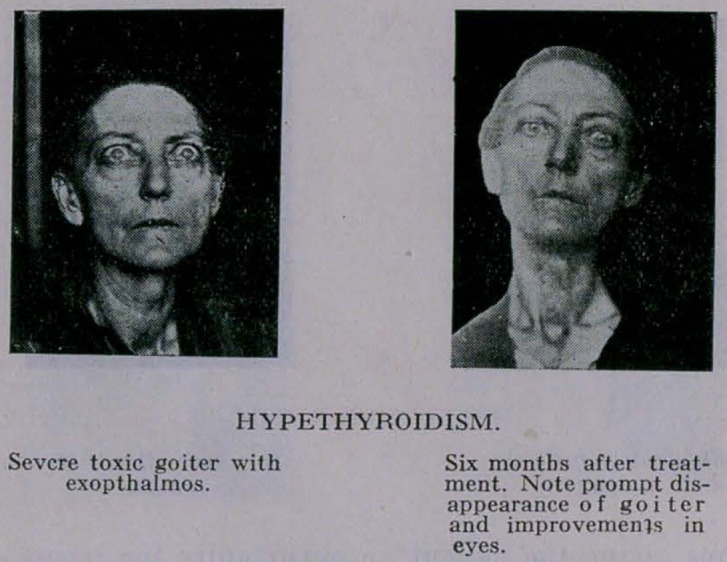# Treatment of Certain Types of Goitre with Quinine and Urea Injections

**Published:** 1916-10

**Authors:** L. F. Watson

**Affiliations:** Oklahoma City


### Treatment of Certain Types of Goitre With Quinine and Urea Injections.

BY L. F. WATSON, M. D., OKLAHOMA CITY.

Assuming that the symptoms of toxic goitre are caused by excessive activity of the thyroid function, many have attempted to limit this secretion by removing a portion of the gland, or ligating its arteries. Approaching the problem from another aspect, some have attempted to neutralize this hyperactivity by means of milk, blood, or serum of animals whose thyroids have been removed; still others have employed injections of different substances into the thyroid. Although these various methods are possessed of advantages and shortcomings, until a longer period of time has elapsed since any of them were instituted, it will be impossible to know just how much value they possessed. There is the question as to the future condition of the patient who has been treated by any of them.

Surgeons and internists agree that the best results follow the thyroid operation when it is performed before the disease has reached the more serious secondary stage—just as a smooth and comfortable recovery will follow the proper medical treatment when administered to beginning cases. If the surgeon is to operate on all favorable cases of beginning hyperthyroidism, surely much useless surgery will be done. Anyone with experience in the disease cannot doubt the value of thyroidectomy as a therapeutic procedure, and in many cases is the only treatment from which the patient may derive benefit. It is my opinion, however, that only in exceptional cases should it be the first step taken to effect a cure. The mortality is high; the recurrence is frequent; and until a greater number of patients have been cured by it, and until a longer period of time has elapsed since it came into use, there will always be the question as to whether the patient operated upon may not suffer at a later time from too little thyroid function. It is well known that thyroidectomy lowers a patient's resistance to disease and infection.

Plummer, from a study of several thousand cases of hyperplastic and colloid goitre, concludes that the disturbances are due to a change in the normal function. The stimulating effect is active

1

2

No. 1.—Human exopthalmic goiter after quinin and urea injection. Note beginning degeneration of epithelium and stroma.

No. 2.—Human exopthalmic goiter after quinin and urea injection. Area of complete necrosis of epithelium and stroma. No connective tissue formation.

throughout the body, and the stimulating action is intra-cellular. These observations have been confirmed by the work of Kendall, who has isolated a crystalline substance containing 60 per cent iodine, and possessing the physiologic activities of the gland.

Bearing in mind these pathological changes which accompany exophthalmic goitre, it is obvious that medical treatment which stops short of destroying a portion of the enlarged and hyperactive gland, will at times fail to afford relief from the acute symptoms, and will also fail to prevent recurrence when the hypersensitive although quiescent goitre is subjected to severe psychic strain.

### DISTURBANCES IN OTHER DUCTLESS GLANDS.

Too often toxic goitre is regarded as a disease of the thyroid gland alone, while in reality all the glands of internal secretion are more or less involved. I have found glycosuria in 85 per cent

HYPERTHYROIDISM.

Moderately toxic cystic goiter. — Two years after treatment.

of the severe cases of hyperthyroidism. Dr. Sajous was the first to emphasize the close relationship and interaction existing between the ductless glands in health and disease.

Rautmann (3) who has recently reported the findings from autopsies on patients dying of exophthalmic goitre, states that an enlarged thymus very frequently accompanies hyperthroidism; the suprarenals and ovaries are involved in a majority of cases; the hypophysis, parathyroids and islands of Langerhans are less frequently affected. He states further, that the changes in the thyroid, parathyroid, thymus, and hypophysis are of a hypertrophic hyperplastic nature, while the changes observed in the suprarenals, ovaries, and islands of Langerhans are of a pronounced atrophic hypoplastic type.

TREATMENT.

Too frequently the hyperthyroid patient is not regarded as a sick person. Because his symptoms may not be severe enough to compel him to stay in bed, the physician is liable to be lax in insisting on close medical supervision. Surgeons and internists agree that any procedure for the treatment of hyperthyroidism must be based upon a period of rest, with medical, dietetic, and hygienic measures suited to the needs of the individual case.

The hyperthyroid patient will usually do best away from home, removed entirely from surroundings suggesting mental and physical exertion. Inquiry will frequently disclose some particular factor of work or worry that has contributed to the symptoms or perhaps caused the disease, and which should be corrected as far as possible. Sympathetic friends and relatives should be ex-

HYPERTHYROIDISM.

Moderately toxic cystic goiter.

One year after treatment.

cluded, thus giving the patient an opportunity for repose, as complete as can be, in a cheerful atmosphere.

ORGANOTHERAPY.

Organotherapy has an established place in the treatment of hyperthyroidism. It is necessary to make a careful study and examination of each patient to determine which ductless glands are contributing to the symptoms, and if their action is one of hypofunction or hyperfunction.

*Hygienic.*—A patient suffering from severe hyperthyroidism should have a rest of several weeks, on an open veranda or in a bright, cheerful room, carefully isolated from seriously ill or noisy patients. The more favorable the climatic conditions, the better for him.

*Dietetic.*—Body weight can be increased best by a simple nourishing diet, with plenty of carbohydrates, milk, fats, vegetables, and a little meat.

*Medicinal.*—The administration of drugs is usually not necessary and should be avoided as far as possible; the treatment of symptoms as they arise is the best rule rather than any routine medication.

QUININE AND UREA INJECTIONS.

In selected cases, I believe hyperthyroidism can be relieved by means of injections of concentrated solutions of quinine and urea into the thyroid.

The method is recommended only to relieve hyperthyroidism and not to remove the goitre. It is sometimes true that in small toxic and atoxic goitre the inflammatory reaction following the injec-

HYPETHYROIDISM.

Severe toxic goiter with exopthalmos.

Six months after treatment. Note prompt disappearance of goiter and improvemenţs in eyes.

tion is sufficient to cause the disappearance of the tumor; but the process is slow, and when the injection is used for this purpose alone, the results are liable to be disappointing.

The procedure is one that is surrounded by certain dangers, immediate and remote. One inexperienced is liable to puncture the trachea or one of the large blood-vessels, or to make the injection into the soft tissues of the neck. Injections that are too extensive will produce the same symptoms of myxedema that follow the removal of too much thyroid by operation. For this reason it is necessary to discontinue injections before symptomatic relief is secured.

The necessity of minimizing the slight pain from any injection by the use of local anesthesia cannot be too strongly emphasized.

Preliminary injections into the thyroid gland of a few minims of a sterile salt solution, followed by injections of sterile water, are necessary to raise the patient's threshold to stimuli, thereby preventing an acute attack of hyperthyroidism which might otherwise follow the slight pain of the first quinine and urea infiltration. As soon as no hyperthyroid reaction follows the water injections, their usefulness is at an end. The use of quinine and urea injections without this preliminary precaution is likely to be disappointing if it is not disastrous.

CONCLUSIONS.

1. The treatment is suggested only to relieve the symptoms of hyperthyroidism and is not recommended to remove the tumor in simple goitre. The method is suitable for use only in a hospital by men experienced in the difficulty of thyroid surgery. The danger of destroying too much of the gland must always be borne in mind.

2. The injection treatment depends just as much as any other procedure for the relief of hyperthyroidism upon the important factor of rest, with careful hygienic and dietetic supervision.

3. The rôle of other ductless glands in contributing to the symptoms of hyperthyroidism must be ascertained and treated accordingly.

4. If the injections are always made well within the thyroid there will be no adhesions around the gland and operation will not be made more difficult because of the previous treatment.

5. Small infiltrations frequently repeated, are to be preferred to massive ones.

6. The injection of a weak solution of quinine and urea has little effect, necrosis and connective tissue formation always occurring after the concentrated solutions are injected.

7. The use of injections of iodine, carbolic acid, alcohol, arsenic, iodoform and chromic acid are to be condemned because of their poisonous and corrosive properties and the liability of producing a thrombus if accidentally injected directly into the blood stream.

8. In treating toxic goitre the necessity of preventing pain from any injection, by the use of local anesthesia, is of vital importance; if acute attacks of hyperthroidism are to be prevented, the use of preliminary injections into the most prominent portion of the goitre of a few minims of sterile salt solution, given at one to three day intervals, followed by injections of sterile water, will be found indispensable; the result of the quinine and urea injection depends on the amount of tissue destroyed.

9. I believe the greatest field of usefulness for the injection will be found in those cases of beginning hyperthyroidism not severe enough to justify operative treatment, and as a preparatory measure to partial thyroidectomy in chronic cases of toxic goitre too ill to warrant any form of immediate operative procedure.

## Figures and Tables

**Figure f1:**
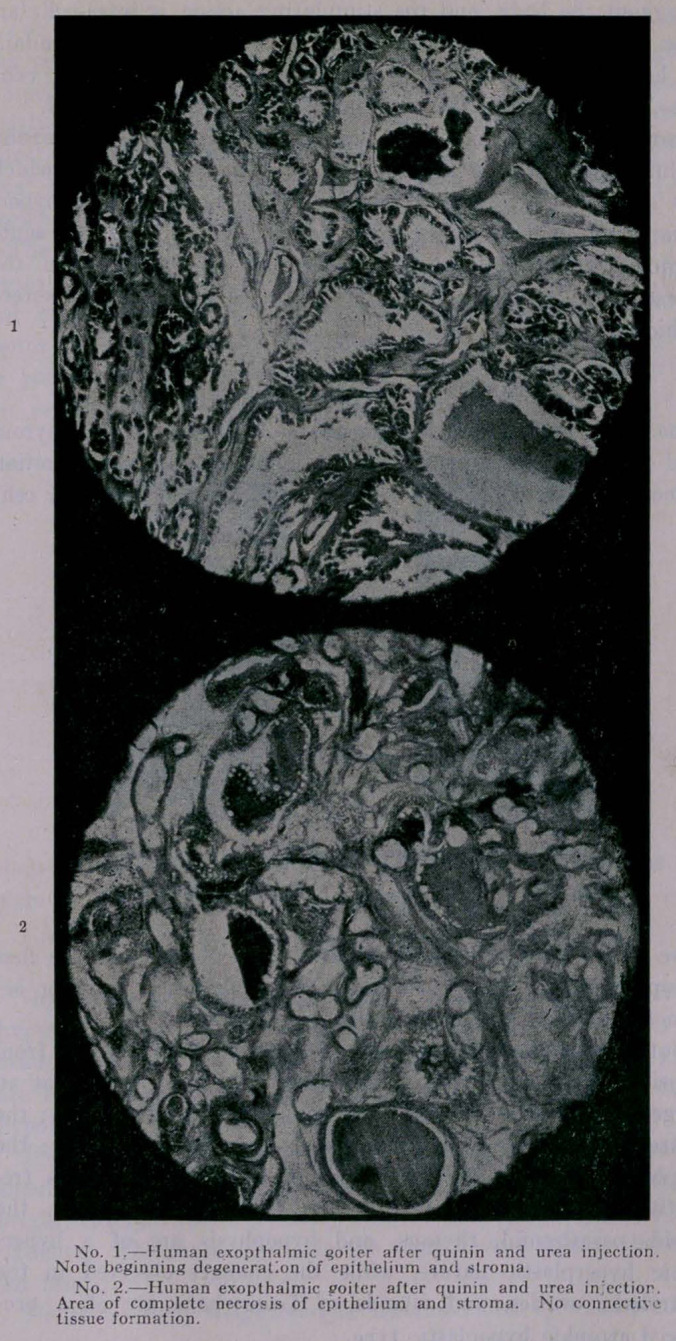


**Figure f2:**
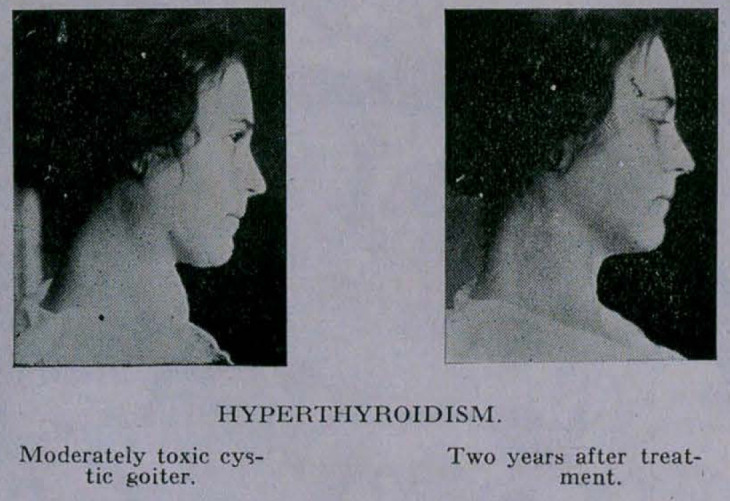


**Figure f3:**
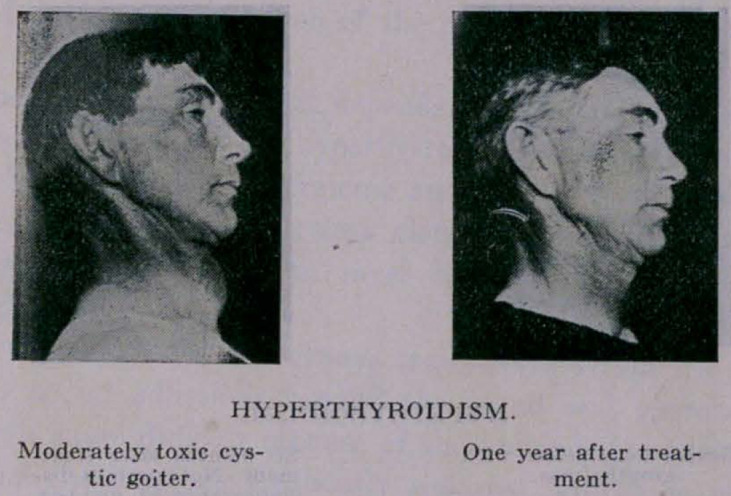


**Figure f4:**